# Gene *sdaB* Is Involved in the Nematocidal Activity of *Enterobacter ludwigii* AA4 Against the Pine Wood Nematode *Bursaphelenchus xylophilus*

**DOI:** 10.3389/fmicb.2022.870519

**Published:** 2022-05-06

**Authors:** Yu Zhao, Zhibo Yuan, Shuang Wang, Haoyu Wang, Yanjie Chao, Ronald R. Sederoff, Heike Sederoff, He Yan, Jialiang Pan, Mu Peng, Di Wu, Rainer Borriss, Ben Niu

**Affiliations:** ^1^State Key Laboratory of Tree Genetics and Breeding, Northeast Forestry University, Harbin, China; ^2^College of Life Science, Northeast Forestry University, Harbin, China; ^3^Administrative Office of the Summer Palace, Beijing Municipal Administration Center of Parks, Beijing, China; ^4^The Center for Microbes, Development and Health (CMDH), Institut Pasteur of Shanghai, Chinese Academy of Sciences, Shanghai, China; ^5^Forest Biotechnology Group, Department of Forestry and Environmental Resources, North Carolina State University, Raleigh, NC, United States; ^6^Department of Plant and Microbial Biology, North Carolina State University, Raleigh, NC, United States; ^7^Center for Biological Disaster Prevention and Control, National Forestry and Grassland Administration, Shenyang, China; ^8^College of Biological Science and Technology, Hubei Minzu University, Enshi, China; ^9^Nord Reet UG, Greifswald, Germany; ^10^Institute of Marine Biotechnology e.V. (IMaB), Greifswald, Germany

**Keywords:** *sdaB*, L-serine dehydratase, *Enterobacter ludwigii*, nematocidal activity, *Bursaphelenchus xylophilus*, pine wilt disease, methuosis

## Abstract

*Bursaphelenchus xylophilus*, a plant parasitic nematode, is the causal agent of pine wilt, a devastating forest tree disease. Essentially, no efficient methods for controlling *B. xylophilus* and pine wilt disease have yet been developed. *Enterobacter ludwigii* AA4, isolated from the root of maize, has powerful nematocidal activity against *B. xylophilus* in a new *in vitro* dye exclusion test. The corrected mortality of the *B. xylophilus* treated by *E. ludwigii* AA4 or its cell extract reached 98.3 and 98.6%, respectively. Morphological changes in *B. xylophilus* treated with a cell extract from strain AA4 suggested that the death of *B. xylophilus* might be caused by an increased number of vacuoles in non-apoptotic cell death and the damage to tissues of the nematodes. In a greenhouse test, the disease index of the seedlings of Scots pine (*Pinus sylvestris*) treated with the cells of strain AA4 plus *B. xylophilus* or those treated by AA4 cell extract plus *B. xylophilus* was 38.2 and 30.3, respectively, was significantly lower than 92.5 in the control plants treated with distilled water and *B. xylophilus*. We created a *sdaB* gene knockout in strain AA4 by deleting the gene that was putatively encoding the beta-subunit of L-serine dehydratase through Red homologous recombination. The nematocidal and disease-suppressing activities of the knockout strain were remarkably impaired. Finally, we revealed a robust colonization of *P. sylvestris* seedling needles by *E. ludwigii* AA4, which is supposed to contribute to the disease-controlling efficacy of strain AA4. Therefore, *E. ludwigii* AA4 has significant potential to serve as an agent for the biological control of pine wilt disease caused by *B. xylophilus*.

## Introduction

The pine wood nematode (PWN) *Bursaphelenchus xylophilus* causes serious damage to forest ecosystems and massive economic losses by inducing pine wilt disease (PWD) (Zhao et al., 2014; Lee et al., 2019; Guo et al., 2020). PWD may result in destruction of conifer forests and has long been a huge threat to Asian and European forestry for a long time (Kikuchi et al., 2011; Faria et al., 2015). As a pathogenic nematode native to North America (Li et al., 2015; Proenca et al., 2017), *B. xylophilus*, feeding on live trees and fungi colonizing dead or dying trees, is a migratory endoparasite transmitted by the insect vector *Monochamus alternatus* (Japanese pine sawyer beetle) (Kim et al., 2019, 2020). The annual economic cost of the PWN (Soliman et al., 2012) in the European Union (EU) alone is estimated at a billion euros for each of the past 22 years. The rapid death of pine trees infected by the PWN could be attributed to the dysfunction of the water-conducting system caused by the death of parenchyma cells. The secreted enzymes and surface coat proteins of *B. xylophilus* are involved in its pathogenicity (Futai, 2013; Nunes da Silva et al., 2015; Wen et al., 2021). The molecular mechanisms of PWN pathogenesis are still largely unknown, hindering the prospects for control of this pathogen and PWD. Chemical insecticides and nematicides used to control *B. xylophilus* by jet-sprays or trunk injections for decades (Qiu et al., 2019; Guo et al., 2020; Faria et al., 2021) have become a major social concern. More environmental-friendly strategies, such as beneficial microorganisms that suppress PWN, have recently gained more attention (Tian et al., 2007; Wu et al., 2013; Cai et al., 2022).

Biological control of plant parasitic nematodes (PPNs) using nematocidal bacteria or their metabolites, which are toxic to nematodes, is a potentially sustainable alternative to chemical nematicides (Haegeman et al., 2009; Kumar and Dara, 2021). Nematocidal prokaryotes mainly belonging to bacterial genera, such as *Bacillus* (Crickmore, 2005; Ponpandian et al., 2019; Park et al., 2020), *Streptomyces* (Kang et al., 2021), *Serratia* (Paiva et al., 2013; Nascimento et al., 2016; Abd El-Aal et al., 2021), *Stenotrophomonas* (Huang et al., 2009; Ponpandian et al., 2019), *Pseudoduganella* (Fang et al., 2019; Abd El-Aal et al., 2021), *Novosphingobium* (Topalović et al., 2020), *Pasteuria* (Tian et al., 2007), *Pseudomonas* (Chan et al., 2020), *Enterobacter* (Munif et al., 2000; Oh et al., 2018), and *Curtobacterium* (Kumar and Dara, 2021), are capable of suppressing PPNs by diverse modes of action, including parasitism (Wu et al., 2013; Proenca et al., 2017; Ponpandian et al., 2019), production of toxins (Abd El-Aal et al., 2021; Kahn et al., 2021), antibiotics plus enzymes (Yang et al., 2007; Huang et al., 2009), competition for nutrients (Proenca et al., 2019), and induction of systemic resistance of plants (Liang et al., 2019; Han et al., 2021). Within these nematocidal groups, some bacterial strains have exhibited efficient killing activity against the PWNs. *Bacillus thuringiensis* zjfc85 caused 90% mortality of *B. xylophilus* by producing a Cry protein named Cry5Ba3 (Kahn et al., 2021). Two *Streptomyces* strains did kill the hatched PWNs and affected egg hatching via biosynthesis of the toxic compounds, teleocidin B4, and spectinabilin, which effectively suppressed the development of PWD under field conditions (Kang et al., 2021). A 70 kD serine protease produced by *Serratia* sp. A88copa13 was majorly responsible for the toxicity of this PWN-killing strain (Paiva et al., 2013). Thus, the application of nematocidal bacteria is a promising strategy in suppressing PWD.

*Enterobacter* is an exceptionally diverse genus of bacteria found in various habitats in association with soil (El-Sayed et al., 2014; Habibi et al., 2019; Danish et al., 2020), plants (Park et al., 2015; Andres-Barrao et al., 2017; Sarkar et al., 2018), and animals, including humans (Mokracka et al., 2004; Peng et al., 2009). Many *Enterobacter* strains are characterized as plant beneficial bacteria. Such procaryotic microbes promote the growth of their plant hosts under favored or adverse abiotic conditions by producing indole-acetic acid (IAA) (Park et al., 2015; Srisuk et al., 2018; Habibi et al., 2019; Li et al., 2022), siderophores (Mokracka et al., 2004; Nurjadi et al., 2021; Li et al., 2022), hydrocyanic acid (Mpongwana et al., 2016; Mahdi et al., 2020; Javaheri Safa et al., 2021), salicylic acid (Kang et al., 2015) plus exopolysaccharides (Sayyed et al., 2015; Niu et al., 2018; Dhanya et al., 2021), solubilizing phosphate (Adhikari et al., 2020; Roslan et al., 2020; Aeron et al., 2021), by fixing nitrogen (Kämpfer et al., 2005; Peng et al., 2009; El-Sayed et al., 2014), reducing Na^+^ uptake (Um et al., 2017; Sarkar et al., 2018), and inducing the activity of enzymes with action as antioxidants (Andres-Barrao et al., 2017; Danish et al., 2020). At present, it has been demonstrated that some *Enterobacter* species exhibit inhibitory effects against the oomycete pathogen *Pythium ultimum* (Lohrke et al., 2002; Kageyama and Nelson, 2003; Windstam and Nelson, 2008), the fungal pathogens *Fusarium moniliforme* (Hinton and Bacon, 1995; Demirci et al., 2000; Rodrigues et al., 2018), *F. oxysporum* (El-Sayed et al., 2014; Del Barrio-Duque et al., 2019; Abdelshafy Mohamad et al., 2020), *Aspergillus niger* (Yadav et al., 2016; Kaushik et al., 2017; Batyrova et al., 2020), and *Aspergillus flavus* (Etcheverry et al., 2009; Mahmood et al., 2020), and the bacterial pathogen *Ralstonia solanacearum* (Sarkar and Chaudhuri, 2015; Yin et al., 2020; Zaki et al., 2021), indicating their protective efficacy on plants. A few *Enterobacter* strains exhibited significant nematocidal activity against the root-knot nematodes (Munif et al., 2000; El-Sayed et al., 2014; Oh et al., 2018). *Enterobacter asburiae* HK169 was able to reduce root gall formation rate by 66% and killed all juveniles of *Meloidogyne incognita* within 48 h (Oh et al., 2018). Similarly, an endophytic *E. intermedius* strain isolated from tomato roots remarkably decreased the number of root galls (Munif et al., 2000). Our knowledge of the molecular mechanisms underlying the inhibitory effects of *Enterobacter* strains against PPNs is still very limited.

In the present study, we identified a powerful PWN-killing bacterial strain, *Enterobacter ludwigii* AA4, which was previously isolated from maize roots (Niu et al., 2017) by screening the nematocidal activities of 374 bacterial strains against *B. xylophilus* using a new fluorescent staining-based PWN-killing activity test. *E. ludwigii* AA4 had a remarkable inhibitory effect against PWD under greenhouse conditions. After deleting the *sdaB* gene, presumed to encode the beta subunit of L-serine dehydratase, the nematocidal and disease-suppressing effects of the mutant strain were significantly reduced. The robust colonization of *P. sylvestris* seedling needles by *E. ludwigii* AA4, presumed to contribute to the disease-controlling efficacy of strain AA4, was quantified and compared to the strain carrying a *sdaB* gene deletion.

## Materials and Methods

### Microbial Strains and Growth Conditions

Bacterial strains kept frozen with 15% (v/v) glycerol at −80°C were streaked on Luria-Bertani (LB) agar plates and incubated at 30°C for 16 h. Then, a single colony of each strain was inoculated into 5 ml LB liquid medium and shaken at 30°C, 200 rpm for another 16 h. Antibiotics were supplemented where necessary at the following concentrations: kanamycin 50 μg/ml, tetracycline 50 μg/ml, gentamicin 25 μg/ml, streptomycin 50 μg/ml, and gentamicin 25 μg/ml (Table 1).

**TABLE 1:** Strains and plasmids used for genetic manipulation of *Enterobacter ludwigii* AA4.

Strain or plasmid	Description
**Strains**	
T1	Tn10 transposon inserting into genome of wild-type AA4; *Kan^R^*.
AA4Δ*sdaB*	Derivative of wild-type AA4 that lacks *sdaB*; *Kan^R^*.
CΔ*sdaB*	AA4Δ*sdaB* complemented with wild-type *sdaB* gene; *Kan^R^*, *Tet^R^*.
AA4-GFP	Green fluorescent protein (GFP)-labeled wild-type AA4; *Tet^R^*, *Gen^R^*.
AA4Δ*sdaB-*GFP	GFP-labeled AA4Δ*sdaB*; *Kan^R^*, *Tet^R^*, *Gen^R^*.
CΔ*sdaB-*GFP	GFP-labeled CΔ*sdaB*; *Kan^R^*, *Tet^R^*, *Gen^R^*.
DH5a	Competent cells for cloning.
**Plasmids**	
pKD4	Kanamycin cassette; *Kan^R^*.
pKD46	Template plasmid containing Red recombinase system under arabinose-inducible promoter; *Kan^R^*.
pUC19	Vector for construction of recombinant plasmid; *Kan^R^*.
pBBR1	Vector for construction of complement plasmid; *Tet^R^*.
pGPF78	Vector with green fluorescent protein (GFP); *Tet^R^*, *Gen^R^*.

*Kan^R^, kanamycin resistance; Tet^R^, tetracycline resistance; Gen^R^, gentamicin resistance.*

The spores of *Botrytis cinerea* were deposited at −80°C in 15% (v/v) glycerol. Ten microliters of fungal spores were dropped on a potato dextrose agar (PDA) plate and incubated at 25°C in the dark. When *B. cinerea* mycelia covered PDA plates, the fungal culture was used for inoculating *B. xylophilus*.

### The Source and Culture of Pine Wood Nematodes

Cultures of the PWN, *Bursaphelenchus xylophilus*, were obtained from Dr. Hongtao Li at the Hebei Academy of Agriculture and Forestry Sciences and Dr. Kai Guo at Zhejiang A&F University. The fungus *B. cinerea* was purchased from the Shanghai Bioresource Collection Center (SHBCC).

*Bursaphelenchus xylophilus* was inoculated on a PDA culture of *B. cinerea* and incubated at 25°C in the dark until the fungal mycelia were completely consumed by *B. xylophilus*. Nematodes were collected using the modified Baermann funnel technique (Kitazume et al., 2018; Cesarz et al., 2019; Maehara et al., 2020) and washed with a mixture of 0.1% streptomycin sulfate and 0.002% actinone three times to remove surface microbial contaminants (Liu et al., 2016). Then, these nematodes were used for PWN-killing activity test and *in planta* biocontrol assays.

For microscopic image analysis of PWN morphology, about 10,000 nematodes were decanted into a burette containing 25 ml of 0.3% carboxymethyl cellulose (CMC) solution. After 12 h, the second-stage juveniles were collected from the top of the burette (Qiu et al., 2016, 2019). These worms were fed with *B. cinerea*. After 48 h, the L4 juveniles were collected in sterile water and washed with a mixture of 0.1% streptomycin sulfate and 0.002% actinone three times. These L4 juveniles were used for the image analysis.

### Preparation of Cell Extracts of *E. ludwigii* AA4

Wild-type AA4, AA4Δ*sdaB* (derivative of wild-type AA4 that lacks *sdaB*), and C△*sdaB* (AA4Δ*sdaB* complemented with wild-type *sdaB* gene) kept frozen with 15% (v/v) glycerol at −80°C were streaked on LB agar plates with corresponding antibiotics (Table 1) and incubated at 30°C for 16 h. Then, a single colony of each strain was inoculated into 5 ml LB liquid medium and shaken at 30°C, 200 rpm for another 16 h. One milliliter of the culture of each strain was transferred into 50 ml LB liquid medium and shaken at 30°C, 200 rpm for 2–3 h until Optical Density at 600 nm (OD_600_) value reached 0.6–0.8 (early stationary phase). Cells were harvested by centrifuging at 9,100 rpm (8,000 × *g*) at 4°C for 20 min (Thermo, Multifuge X1R, Germany) and washed by phosphate-buffered saline (PBS). The cell precipitations were resuspended in 5 ml PBS before sonication. An ultrasound processor (Sonics, VCX130PB, United States) was used to sonicate the resuspended cell suspensions. The probe was immersed into the suspensions and sonicated the cells on ice for 60 min at a power of 130 W (pulse duration: 2 s on, 1 s off). The resulting cell lysates were centrifuged at 15,300 rpm (14,000 × *g*) for 30 min. The supernatant was collected and used as cell extracts. The concentration of total protein in cell extract was measured by using a biuret protein assay reagents kit (Solarbio, PC0010, China).

### Fluorescent Staining-Based Pine Wood Nematode-Killing Activity Test

The nematocidal activity of the 43 efficient PWN-killing bacterial strains of AA4Δ*sdaB* and CΔ*sdaB* was evaluated by the fluorescent staining-based PWN-killing activity test, while *Bacillus pumilus* YLT40 and *Paenibacillus polymyxa* M-1 were utilized for confirming the reliability of the assay. The cell density of bacterial culture suspension was adjusted to OD_600_ = 1.0 for each strain used in the test. Each well of the 96-well microplates was added with 40 μl of bacterial culture suspension and 20 μl of worm suspension containing 40–50 nematodes, while 40 μl of LB liquid medium or PBS and 20 μl of worm suspension was employed as negative control. Five wells were used for each treatment designated with the code of the strain listed in Supplementary Tables 1, 2. The inoculated microplates were incubated at 25°C in the dark for 24 h. Then, a plate washer (Biobase, BK-9622, China) was employed to change the liquid cultures with sterile water. 4’6-diamidino-2-phenylindole (DAPI, Solarbio, C0065, China) was used to stain the PWNs, and the final concentration of DAPI in each well was 5 μg/ml. After being stained for 1 h, the nematodes were observed by a confocal laser scanning microscope (Zeiss, LSM 800, Germany) using a excitation laser of 353 nm and collecting emission of 465 nm, where the dead worms displayed bright fluorescence while the live ones were dim.

To test the nematocidal activity of the cell extract of wild-type AA4, AA4Δ*sdaB*, and CΔ*sdaB*, the extract containing total proteins of 5 mg/ml was mixed with 20 μl of worm suspension containing 40–50 nematodes in one well of a 96-well microplate. The remaining steps are the same as those for the assays with bacterial cell suspensions.

The mortality and corrected mortality of *B. xylophilus* were calculated as follows (Faria et al., 2013; Guo et al., 2016; Liu et al., 2019):


$$\mathrm{The}\mathrm{mortality}\left(\%\right)=\frac{\mathrm{number}\,\mathrm{of}\,\mathrm{dead}\,\mathrm{nematodes}}{\mathrm{number}\,\mathrm{of}\,\mathrm{all}\,\mathrm{nematodes}}\times 100$$

$$\mathrm{Corrected}\mathrm{mortality}\left(\%\right)=\frac{\mathrm{mortality}\mathrm{in}\mathrm{treatment}\left(\%\right)-\mathrm{mortality}\mathrm{in}\mathrm{control}\left(\%\right)}{100-\mathrm{mortality}\mathrm{in}\mathrm{control}\left(\%\right)}\times 100$$

Two-tailed *t*-test (GrapPad Prism 8) was used for the statistical analysis. All these experiments above were repeated five times.

Z′factor, a combination of signal interval and variation, is a parameter used to evaluate the reliability of results obtained from an experiment for guiding a larger-scale experimentation. It is calculated as:


z⁢factor′=1-3⁢(σ⁢p+σ⁢n)|μ⁢p-μ⁢n|

where σ_*p*_ and σ_*n*_ represent the standard deviations of positive control and negative control, respectively, and μ_*p*_ and μ_*n*_ represent the means of positive control and negative control, respectively. Z′factor was calculated by using the software Excel (Microsoft). The theoretical value of Z′factor is 1, and a Z′factor value between 0.5 and 1 indicates the outcomes of a given experiment is highly reliable, while a test of low reliability usually possesses a Z′factor value of less than 0.5 (Zhang X. D. et al., 2020).

### Identification of the Efficient Bacterial Pine Wood Nematode-Killing Strains

Identification of the efficient bacterial PWN-killing strains were carried out by using 16S rRNA gene sequencing analysis. Genomic DNA was extracted from an overnight LB culture of each strain by utilizing a bacterial genomic DNA extraction kit (Tiangen, DP302-02, China) following the manufacturer’s protocol. The 16S rRNA gene was amplified in 25-μl PCR reactions using the universal primers 27F and 1492R (Table 2) (Monciardini et al., 2002; Niu et al., 2013; López and Alippi, 2019). The reaction mixture contained 1 μl of template DNA, 12.5 μl of master mix (Takara, RR350Q, Japan), 1 μl of each of the forward and reverse primers, and 9.5 μl of sterile deionized water. Amplifications were performed using a T100 Thermal Cycler (Bio-Rad, 621BR47532, United States) with the following cycle conditions: initial denaturation at 95°C for 5 min; 30 cycles at 94°C for 30 s, 58°C for 30 s, and 72°C for 1.5 min; and a final extension at 72°C for 10 min. Amplicons were purified using a PCR purification kit (Omega, D6492-02, China) and sequenced using 27F primer at Tsingke Biotechnology Co., Ltd. DNA sequences were inspected for base-caller errors and were trimmed by removing any ambiguous trailing or leading bases using the software GAP4 in the STADEN Package^1^. The sequences were compared with those of the reference organisms by Basic Local Alignment Search Tool (BLAST) at the National Center for Biotechnology Information (NCBI) website^2^ and by Sequence Match at the Ribosomal Database Project (RDP) website^3^. The 16S rRNA gene sequences of efficient nematocidal strains were deposited in the GenBank database. The accession numbers were listed in Supplementary Table 1.

**TABLE 2:** Primers used in this study.

Primer	5′–3′ sequence
**For 16S rRNA gene sequencing analysis**	
27F	AGAGTTTGATCATGGCTCAG
1492R	TACGGTTACCTTGTTACGACTT
**For gene deletions**	
*sdaB* -H1up	TCTGATTCCGCTGATCATCATGGCTATCATTGCCTTC
*sdaB* -H1down	GTAATGCTGCAATCTGATGCGTCCATTGCTTTCAGTCAGAGGGGGAGGAG
*sdaB* -H2up	GAAGAAGCCTCGCATAACGAGGCTTCCTGAAAGGCATATCCTCCTTAGTTCCTATTCC
*sdaB* -H2down	GTGCGATATGGTTGAGAAGCCAGCAAAAGTGGCC
Kup	GGCTTCCTGAAAGGCATATCCTCCTTAGTTCCTATTC
Kdown	AGCAATGGACGCATCAGATTGCAGCATTACACGTCTT
pUC19up	CATGATGATCAGCGGAATCAGATCGCGCGTTTCGGTGATGACGGTGAAAACCTCTGAC
pUC19down	CACTTCTTCGCGGTCGTGATACGACGAAAGGGCCTCGTGATACGCCTATTTTTATAGG
**For complementing the mutation**	
*sdaB*-DF	ATGGAAACCACTCAAACCAGCACCGTTGCTTCGATTG
*sdaB*-UR	CGAGCTGCGTGTTGAAGTCGCGGATACCAACGAAAGC
*sdaB*-PBBR1-DF	AAGACAGAATCAGAATtCAATTCGCCCTATAGTGAGTCGTATTA
sdaB-PBBR1-UR	TTGTGACCTGCGATTAACAGCTTTTGTTCCCTTTAG

### *In planta* Assays for Biocontrol Effect Against Pine Wilt Disease

The biological control effects of wild-type AA4, AA4Δ*sdaB*, and CΔ*sdaB* were examined by using both 3-year-old and 1-month-old Scots pine (*Pinus sylvestris*) tree seedlings grown under greenhouse conditions. The 3-year-old seedlings were purchased from Longsheng nursery at Harbin. Ten milliliters of bacterial suspension (OD_600_ = 1.0) were inoculated on the 3-year-old seedlings by spraying. Then, a 2–4 cm silt was made on the surface of bark located at approximately 10–15 cm above the soil. One hundred microliters of PWN suspension containing about 5,000 worms were injected into the trunk. A small piece of sterilized cotton was fixed on the trunk to cover the silt (Xue et al., 2019). The plants were placed in a greenhouse under the following conditions: 16 h of light (day) and 8 h of dark (night), 25°C, and a relative humidity of 70%. Plants were watered periodically and maintained in the greenhouse. Thirty days after inoculation, the symptoms present on the seedlings were recorded with a camera (Canon, 80D, Japan). The severities of PWD were evaluated based on the disease ranks described in earlier studies (Qiu et al., 2016, 2019) as follows: In rank 0, all needles are green. In rank 1, less than a quarter of needles turn yellow. In rank 2, 25–75% of needles turn yellow. In rank 3, more than 75% of needles turn yellow and less than 50% of needles get wilted. In rank 4, more than 50% of the needles get wilted. Five plants were used for each of the two treatments designated as PWN (inoculation of seedlings with PWN alone) and PWN + AA4 (inoculation of seedlings with PWN jointly with wild-type AA4), respectively.

For the experimentations with 1-month-old *Pinus sylvestris* seedlings, the seeds were surface-sterilized by immersing in 5% KMnO_4_ for 60 min and placing in a growth chamber at 30°C in the light for 48 h. After germination, the seeds with roots were sown in the plant growth substrates prepared by mixing soil and vermiculite in a ratio of 3:1. Thirty days after emergence, 5 ml of bacterial suspensions (OD_600_ = 1.0) or cell extracts (containing total proteins of 5 mg/ml) of wild-type AA4, AA4Δ*sdaB*, and CΔ*sdaB* were sprayed on the needles of Scots pine seedlings, respectively. In the meantime, 100 μl of PWN suspension containing approximately 3,000 worms were injected into the needles by using a syringe. A small piece of sterile cotton was fixed on the needles to cover the pinhole. The plants were incubated in greenhouse under the same condition on which the 3-year-old seedlings grew. The number of dead seedlings and symptoms were recorded on the ninth day after inoculation. Ten plants were used for each of the 11 treatments designated as PWN (inoculation of seedlings with PWN alone), PWN + Δ*sdaB* (inoculation of seedlings with PWN jointly with AA4Δ*sdaB* cell suspension), PWN + CΔ*sdaB* (inoculation of seedlings with PWN jointly with AA4CΔ*sdaB* cell suspension), PWN + WT (inoculation of seedlings with PWN jointly with wild-type AA4 cell suspension), PWN + Δ*sdaB*CE (inoculation of seedlings with PWN jointly with AA4Δ*sdaB* cell extract), PWN + CΔ*sdaB*CE (inoculation of seedlings with PWN jointly with AA4CΔ*sdaB* cell extract), PWN + WTCE (inoculation of seedlings with PWN jointly with wild-type AA4 cell extract), PWN + Δ*sdaB*S (inoculation of seedlings with PWN jointly with AA4Δ*sdaB* culture supernatant), PWN + CΔ*sdaB*S (inoculation of seedlings with PWN jointly with AA4CΔ*sdaB* culture supernatant), PWN + WTS (inoculation of seedlings with PWN jointly with wild-type AA4 culture supernatant), and DW (treatment with distilled water), respectively.

The severities of PWD were evaluated based on the disease ranks shown in Supplementary Figure 3. The disease severity indices were calculated as follows:


$$\mathrm{Disease}\,\mathrm{Index}\,\left(\text{DI}\right)=\frac{\sum \left(\text{Xi}\times \text{Ai}\right)}{\left(\text{X}\times \text{Amax}\right)}$$

where Xi is the number of pine trees in each disease rank, Ai is the disease rank, X is the total number of pine trees, and Amax is the maximum rank (Qiu et al., 2019; Xue et al., 2019). Two-tailed *t*-test (GrapPad Prism 8) was used for the statistical analysis. These experiments were repeated three times.

### Image Analysis of Pine Wood Nematode Morphology

The cell extract of *E. ludwigii* AA4 was prepared as above. Forty microliters of cell extract containing total proteins of 5 mg/ml and 20 μl of worm suspension containing 40–50 L4 juveniles of PWNs were added in a single well of the 96-well microplates. At the meantime, the combination of 40 μl of PBS and 20 μl of worm suspension was employed as negative control. The microplates were stored at 25°C in the dark for 24 h. Then, the worms were picked and put into 10 μl of PBS by injector pinhead. After being washed three times, the worms were transferred to a slide. Ten microliters of PBS were then dropped on the samples. The morphological changes of *B. xylophilus* were observed and recorded by a light microscope (Olympus, BX43, Japan).

### Construction of Mutants of *E. ludwigii* AA4

The *sdaB* gene knock-out mutant of *E. ludwigii* AA4 was created by using the Red recombinase system (Datsenko and Wanner, 2000; Dahyot et al., 2020; Guérin et al., 2020). Primers were designed according to the information obtained from the whole genome sequence of strain AA4 (Niu et al., 2017). The upstream border sequence of the AA4 *sdaB* gene was amplified from AA4 chromosomal DNA using primers *sdaB*-H1up and *sdaB*-H1down (Table 2), while the downstream border sequence of *sdaB* was amplified with primers *sdaB*-H2up and *sdaB*-H2down (Table 2). A kanamycin resistance cassette, flanked by 23-bp-long sequences homologous with *sdaB* gene, was amplified from plasmid pKD4 DNA with primers Kup and Kdown (Table 2). Another fragment flanked by 22-bp-long sequences homologous with *sdaB* gene was amplified from plasmid pUC19 by using primers pUC19up and pUC19down (Table 2). The purified PCR products were assembled by Gibson DNA assembly technology, using a SoSoo Cloning Kit (Tsingke, T-TSV-S1, China) following the manufacturer’s instructions and then cloned into *Escherichia coli* DH5α. Clones containing the resulting recombinant pU19Δ*sdaB* vector were selected on LB agar supplemented with kanamycin. The recombinant plasmid pUC19Δ*sdaB* was isolated with a plasmid extraction kit (Omega, D6943-02, China) following the manufacturer’s instructions and used as template DNA for PCR amplification with the primers *sdaB*-H1up and *sdaB*-H2down (Table 2). After purification, the PCR products were introduced into the competent cells of AA4 carrying a Red helper plasmid pKD46 by electroporation (Bio-Rad, 411BR11661, United States) at 2.5 KV. The resulting transformants were selected on LB agar supplemented with kanamycin after incubation for 24 h at 30°C. Homologous recombination was confirmed by PCR and sequencing. Then, the elimination of pKD46 was performed by culturing the correct transformant by shaking at 37–42°C, 200 rpm overnight. The knock-out mutant can only grow at 30°C on the LB agar supplemented with kanamycin but not streptomycin.

To construct a complement strain of AA4Δ*sdaB*, the complete open reading frame of *sdaB* gene was amplified by PCR with primers *sdaB*-DF and *sdaB*-UR (Table 2) from the genomic DNA of strain AA4 and linked with the fragment flanked by 22-bp-long sequences homologous with *sdaB* gene and amplified from plasmid pBBR1 using primers *sdaB*-PBBR1-DF and *sdaB*-PBBR1-UR (Table 2) by the Gibson DNA assembly technology. The complement plasmid pBBR1*-sdaB* was introduced into the competent cells of AA4Δ*sdaB by* electroporation at 2.5 KV, and the resulting transformants were selected on LB agar supplemented with kanamycin and tetracycline after incubation at 30°C for 24 h. The complementation was then confirmed by PCR and sequencing.

To create a random transposon library, *E. ludwigii* AA4 was transformed with pDL1093 via conjugation at 30°C and outgrown at 42°C in the presence of kanamycin to induce transposition of miniTn10 (Duncan et al., 2018).

For the sake of constructing fluorescence protein-labeled AA4, plasmid pGFP78, obtained from Dr. Qi Wang at China Agricultural University, harboring the gene encoding for green fluorescent protein (GFP), was introduced into the competent cells of wild-type AA4, AA4Δ*sdaB*, or CΔ*sdaB* by electroporation at 2.5 KV. The resulting transformants were selected on LB agar supplemented with tetracycline, or kanamycin and tetracycline, or a combination of kanamycin, tetracycline, and gentamicin where necessary after incubation at 30°C for 24 h. The correctness of the transformants were then confirmed by PCR, sequencing, and fluorescence microscopy.

### Assay for L-Serine Dehydratase Activity

The cell extract of *E. ludwigii* AA4 was prepared as above, except washing and resuspending in the extraction buffer (50 mM K_2_HPO_4_, 2.5 mM serine protease inhibitors) (Velayudhan et al., 2004). Three aliquots of 1 ml of cell extract containing total proteins of 10 mg/ml was incubated with 500 μl of 300 mM L-serine at 37°C for 6 min. Then, the concentration of pyruvate was determined by using a pyruvate assay kit (Solarbio, BC2205, China). The L-serine hydrolase activity was calculated as:


(1) $$l-\mathrm{serine}\,\mathrm{hydrolase}\,\mathrm{activity}\,\mathrm{nmol}/\min\,\text{⋅}\,\mathrm{mg}\,\mathrm{of}\,\mathrm{protein}=\frac{\frac{\text{c}\times \text{v}}{\text{M}}}{\text{T}\times \text{m}}\times 10^{3}$$

where c is the concentration of pyruvate in μg/ml, v is the total reaction volume in ml, M is the relative molecular mass of pyruvate, T is the reaction time in minutes, and m is the weight of cell extract in mg.

Two-tailed *t*-test (GrapPad Prism 8) was used for the statistical analysis. This experiment was repeated five times.

### Assays for Colonization of Scots Pine Needles

In order to investigate the ability of *E. ludwigii* AA4 to colonize Scots pine needles, 50 ml of LB cultures (OD_600_ = 1.0) of GFP-labeled wild-type AA4, AA4Δ*sdaB*, and CΔ*sdaB* were sprayed on 21 1-month-old seedlings, respectively. The plants were placed in a greenhouse under the following conditions: 16 h of light (day) and 8 h of dark (night), 25°C, and a relative humidity of 70%. Three aliquots of 50 mg of needles were sampled at the 4th, 48th, 72th, 96th, 120th, and 144th, and 168th hour post inoculation, respectively, for bacterial quantification. The needles were crushed with 1 ml of PBS by using a sterile mortar. The resulting suspensions were diluted and spread on LB agar plates supplemented with tetracycline and incubated at 30°C for 16 h. The colony-forming unit (CFU) numbers were recorded. Two-tailed *t*-test (GraphPad Prism 8) was used for the statistical analysis. This experiment was repeated five times.

For image analysis, 48 h after inoculation, the needle slices were washed with PBS and transferred to a slide. Ten microliters of PBS were dropped on the samples. The colonization of needles by bacteria was visualized by a Confocal Laser Scanninc Microscopy (CLSM; Zeiss, LSM 800, Germany) using an excitation laser of 480 nm.

## Results

### Screening of Bacterial Strains for Nematocidal Activity Against *Bursaphelenchus xylophilus*

**FIGURE 1:**
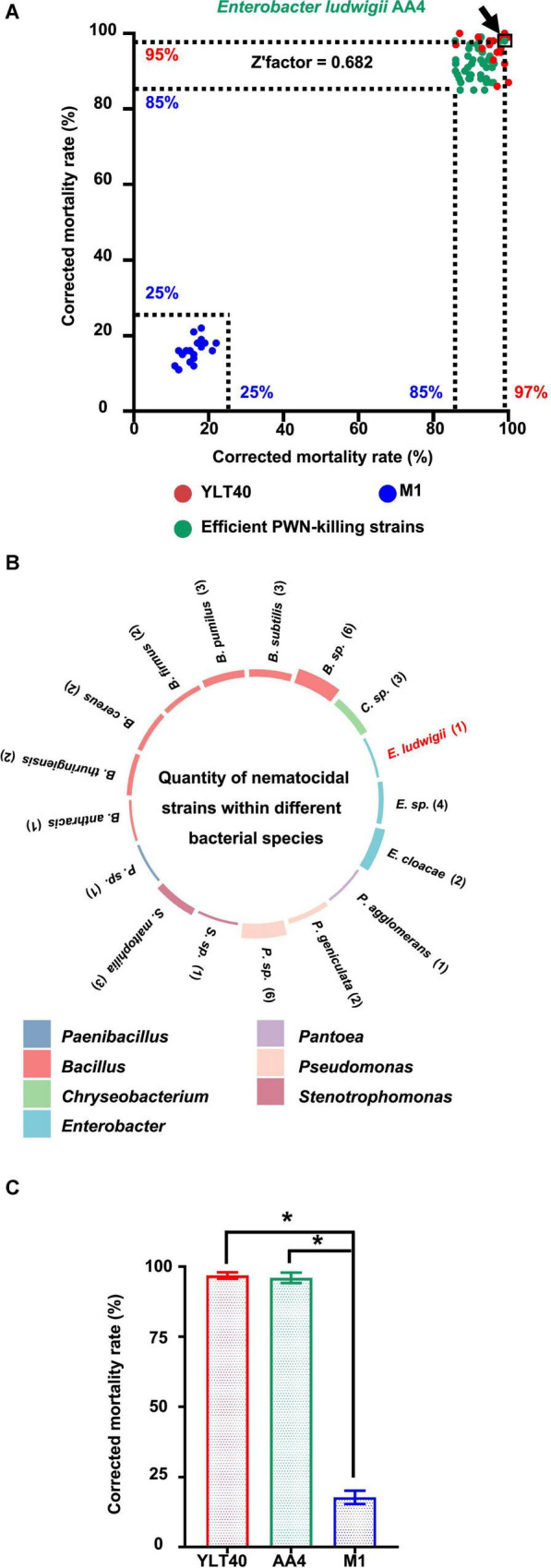
Screening and taxonomy of the nematocidal bacterial strains against pine wood nematodes (PWNs). **(A)** Screening of strong PWN-killing bacterial strains against *Bursaphelenchus xylophilus*. The green dots represent the 43 strong nematocidal strains causing the death of more than 85% of *B. xylophilus* treated with them, while the blue and red dots are bacterial cultures of *Bacillus pumilus* YLT40 and *Paenibacillus polymyxa* M-1 used as controls to confirm the reliability of the screening. The position of each dot was determined by the two values (showing on the *x*- and *y*-axis, respectively) of corrected mortality rate of PWNs calculated in two independent tests. The dot standing for *Enterobacter ludwigii* AA4 is framed and pointed out by an arrow. **(B)** The number of nematocidal strains within each bacterial species. The colors of the columns on the circle indicate the genus names of the 43 nematocidal strains, while histogram heights are consistent with the numbers (showing in the brackets) of the strains belonging to each bacterial species. **(C)** Nematocidal effect of *E. ludwigii* AA4 against PWNs. Asterisks indicate that differences among the means represented by the columns are statistically significant (*p < 0.0001). Two-tailed *t*-test (GrapPad Prism 8) was used for the analysis.

To find microorganisms possessing strong nematocidal activity against *B. xylophilus* for potential biological control of the PWD, we performed a PWN-killing test with 374 bacterial strains from our lab collection. First, we used an efficient PWN-killer *Bacillus pumilus* YLT40 and a moderate PWN-killing strain *Paenibacillus polymyxa* M-1 (Niu et al., 2013), identified previously (data not shown), as controls (Figure 1A) to verify if the PWN-Bacterium interaction system employed in this study was reliable for a large-scale screening. The corrected mortality rates of the nematode *B. xylophilus* treated by *B. pumilus* YLT40 and *P. polymyxa* M-1 were more than 85% and less than 25% (Figure 1A), respectively. We calculated a Z′factor score of 0.682 (Figure 1A) which indicated that the screening procedure utilized is sufficiently reliable for selecting powerful PWN-killing strains.

We detected 43 efficient nematocidal bacterial strains exhibiting *B. xylophilus* mortality rates above 85% (Figure 1A, Supplementary Figure 1, and Supplementary Table 1). Based on 16S rRNA gene sequencing, we found that these bacterial strains belong to 21 species from seven genera, including *Bacillus*, *Pseudomonas*, *Enterobacter*, *Chryseobacterium*, *Stenotrophomonas*, *Pantoea*, and *Paenibacillus* (Figure 1B), all genera with known nematocidal activity. Among the seven genera, *Bacillus* harbored 19 nematocidal strains, while *Pantoea* and *Paenibacillus* possessed only one PWN-killing strain (Figure 1B). Interestingly, seven *Enterobacter* strains, including the plant beneficial bacterium *E. ludwigii* AA4 isolated from maize roots (Niu et al., 2017), exhibited high nematocidal activity against the PWN (Figure 1B). In addition, AA4 did kill almost all of the *B. xylophilus* clones (96%) in our assay (Figure 1C). In our collection, we found 43 strong PWN-killing strains involving *E. ludwigii* AA4. Next, we validated the PWN-killing function of strain AA4 in both *in vitro* and *in planta* experiments.

### Biocontrol Effect of *E. ludwigii* AA4 Against *B. xylophilus*

**FIGURE 2:**
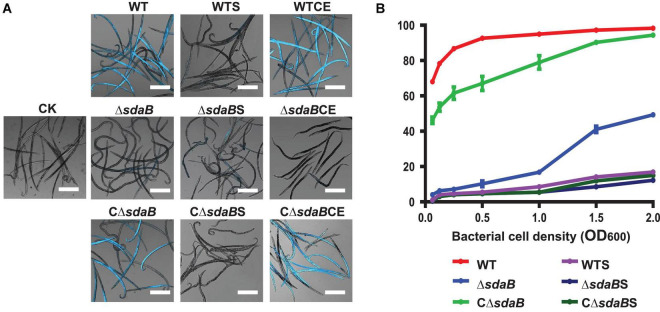
Nematocidal effect of *E. ludwigii* AA4 and its *sdaB* gene knock-out mutant against *B. xylophilus*. **(A)** Imaging analysis of the PWN-killing efficacy of wild-type *E. ludwigii* AA4 and AA4Δ*sdaB* against *B. xylophilus.* The dead nematodes showed cyan fluorescence when excited by 353 nm wavelength after being stained by 4′6-diamidino-2-phenylindole (DAPI), while the live worms stained by DAPI were dim under excitation with the same wavelength. These images were taken using a confocal laser scanning microscope (scale bars: 2 mm). **(B)** Mortality of the PWNs treated with bacterial cells or culture supernatants. *B. xylophilus* was co-cultured with cells of wild-type AA4 and AA4Δ*sdaB* or incubated with culture supernatants of wild-type AA4 and AA4Δ*sdaB* for 24 h, respectively. CK, sterile LB liquid medium; WT, cell culture of wild-type AA4; WTS, supernatant of wild-type AA4 culture; WTCE, cell extract of wild-type AA4; Δ*sdaB*, cell culture of AA4Δ*sdaB*; Δ*sdaB*S, supernatant of AA4Δ*sdaB* culture; Δ*sdaB*CE, cell extract of AA4Δ*sdaB*; CΔ*sdaB*, cell culture of AA4Δ*sdaB* complemented with the wild-type *sdaB* gene; CΔ*sdaB*S, supernatant of culture of AA4Δ*sdaB* complemented with the wild-type *sdaB* gene; CΔ*sdaB*CE, cell extract of AA4Δ*sdaB* complemented with the wild-type *sdaB* gene.

To confirm the nematocidal effect of *E. ludwigii* AA4 against *B. xylophilus*, we developed a fluorescent dye-exclusion viability test to visualize PWN-killing activity. We used DAPI which differentially stains live and dead PWNs. Dead worms take up DAPI rapidly and show bright fluorescence, while the live ones are relatively non-fluorescent and look dim (Figure 2A).

In our assay, the majority (68%) of PWNs co-cultured with wild-type *E. ludwigii* AA4 cells in very low density (OD_600_ = 0.063) for 24 h were killed (Figure 2B). The corrected mortality rate of the nematodes treated with wild-type AA4 cells tends to increase with enhanced cell density. The percentage of dead worms dramatically increased from less than 70% to more than 92% when the OD_600_ value of the bacterial culture was raised from 0.063 to 0.5. The corrected mortality rate rose slowly to a maximum of 98.3% when the OD_600_ value of the cell suspension reached 2 (Figure 2B). Our visual test documented that nearly all the nematodes incubated with wild-type AA4 emitted intense fluorescent signals, while almost no fluorescence was detected without bacteria (Figure 2A). These results well corroborated the high mortality rate of *B. xylophilus* when exposed to wild-type AA4. *E. ludwigii* AA4 exhibited a remarkable *in vitro* PWN-killing effect.

**FIGURE 3:**
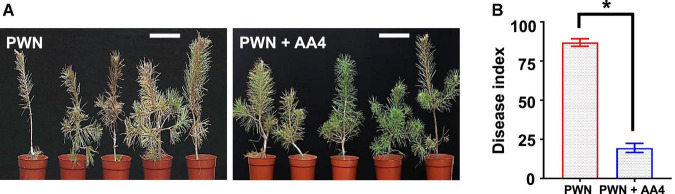
Biocontrol effect of *E. ludwigii* AA4 against PWD caused by *B. xylophilus*. **(A)** Symptoms on the 3-year-old *Pinus sylvestris* seedlings inoculated with PWN alone or PWN jointly with *E. ludwigii* AA4 (PWN + AA4). Photographs were taken 30 days after inoculation (Scale bars: 15 cm). **(B)** Severity of PWD on the 3-year-old *P. sylvestris* seedlings inoculated with PWN alone and PWN jointly with *E. ludwigii* AA4 (PWN + AA4). The asterisk indicates that differences among the means represented by the columns are statistically significant (*p < 0.0001). Two-tailed *t*-test (GrapPad Prism 8) was used for the analysis.

We then investigated the disease-suppressing efficacy of *E. ludwigii* AA4 against PWD on 3-year-old *Pinus sylvestris* greenhouse-grown seedlings. Thirty days after inoculation, we found that the needles of the seedlings treated with PWNs together with wild-type AA4 were much greener and less wilted than those inoculated with *B. xylophilus* only (Figure 3A). The disease index of PWD on the *P. sylvestris* plants treated with PWNs together with wild-type AA4 (19.5) was significantly lower than that treated with *B. xylophilus* alone (86.9) (Figure 3B), indicating that the PWD severity was substantially reduced by treatment with wild-type AA4. *E. ludwigii* AA4 was capable of efficiently suppressing PWNs not only under *in vitro* conditions but also in *in planta* and was a strong bacterial biocontrol strain against PWD caused by *B. xylophilus*.

### *E. ludwigii* AA4 Causes Morphological Defects in *B. xylophilus*

**FIGURE 4:**
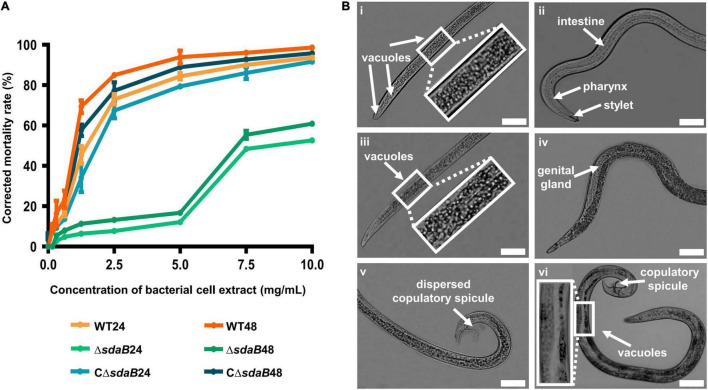
Nematocidal effect of cell extracts of *E. ludwigii* AA4 and its *sdaB* gene knock-out mutant against *B. xylophilus*. **(A)** Mortality of the *B. xylophilus* nematode treated with bacterial cell extracts. The worms were incubated with the cell extracts of wild-type AA4 and AA4Δ*sdaB* for 24 h or 48 h, respectively. WT24, PWNs treated with a cell extract of wild-type AA4 for 24 h; WT48, PWNs treated with a cell extract of wild-type AA4 for 48 h; Δ*sdaB*24, PWNs treated with a cell extract of AA4Δ*sdaB* for 24 h; Δ*sdaB*48, PWNs treated with a cell extract of AA4Δ*sdaB* for 48 h; CΔ*sdaB*24, PWNs treated with a cell extract of AA4Δ*sdaB* complemented with a wild-type *sdaB* gene for 24 h; CΔ*sdaB*48, PWNs treated with a cell extract of AA4Δ*sdaB* complemented with a wild-type *sdaB* gene for 48 h. **(B)** Morphological changes of *B. xylophilus* incubated with the cell extract of wild-type *E. ludwigii* AA4. The L4 stage worms exposed to a wild-type AA4 cell extract containing total proteins of 5 mg/mL for 24h, are shown in the left column, where the vacuoles in front **(i)** and back **(iii)** sections of worm bodies as well as dispersed copulatory spicule **(v)** are indicated by arrows. Non-treated L4 stage nematodes **(ii,iv,vi)** are presented in the right row, where the arrows indicate the intestine, pharynx, and stylet **(ii)**, genital gland **(iv)**, copulatory spicule and vacuoles **(vi)**, respectively (scale bars: 1 mm).

To further understand the PWN-killing effect of *E. ludwigii* AA4, culture supernatants and cell extracts were prepared for the DAPI-staining based nematocidal activity test. Treatment of *B. xylophilus* with the culture supernatant of wild-type AA4 did not affect the mortality rate of the nematode (Figure 2A). Wild-type AA4 cells did dramatically enhance fluorescence of the *B. xylophilus* nematodes (Figure 2A). This result was corroborated by the mortality rate of the PWNs, suggesting that the death rate of worms exposed to wild-type AA4 culture supernatant (less than 20%) was significantly less than that of *B. xylophilus* incubated with cells of wild-type AA4 (more than 60%) (Figure 2B). By contrast, we detected intense fluorescent signals from the worms treated with wild-type AA4 cell extract (Figure 4A) and found that more than 80% of PWNs were dead after being exposed to the extract (Figure 4A), indicating its strong nematocidal effect. Thus, our data indicates that the cell extract of wild-type AA4 rather than the culture supernatant contains the nematocidal activity.

To elucidate the mode of action of the nematocidal effect of *E. ludwigii* AA4 against PWN, we choose its cell extract for investigating morphological variations of AA4-treated *B. xylophilus*. The L4 stage nematodes were incubated with wild-type AA4 cell extract. Afterward, the morphological changes of the worms were observed and recorded using an optical light microscope. Exposure of PWN to cell extract-induced formation of vast amounts of vacuoles filled the internal tissues of nematodes completely (Figures 4Bi,iii). Newly formed small vacuoles accumulate and progressively fuse into giant ones, leading to membrane rupture and death (Maltese and Overmeyer, 2014; Armenta and Dixon, 2020). This process is defined as methuosis (Cai et al., 2013; Chen et al., 2014; Nirmala and Lopus, 2020), a type of non-apoptotic cell death widely described in animals (Jang et al., 2016; Rajasekharan et al., 2017; Song et al., 2021). The death of *B. xylophilus* caused by AA4 might be attributed to methuosis induced by the damage to cell membranes.

In these vacuolated worms, some vital organs, e.g., intestine, pharynx, and genital glands, disappeared in the mass of bubbles. We also failed to capture a clear structure of stylet at the head of PWN treated with wild-type AA4 cell extract (Figures 4Bi,iii). The reproductive organ of male worms, the copulatory spicule, dispersed after being incubated with cell extract (Figure 4Bv). In contrast, we observed fewer vacuoles in the control nematodes exposed only to PBS. All the critical internal organs were intact and clearly visible in the control worm bodies (Figures 4Bii,iv,vi). Therefore, *E. ludwigii* AA4 appeared to destroy crucial internal organs of *B. xylophilus* through causing a series of morphological defects.

### The *sdaB* Gene Is Involved in the Nematocidal Effect of *E. ludwigii* AA4 Against *B. xylophilus*

To resolve the molecular mechanisms underlying the strong nematocidal effect of *E. ludwigii* AA4, we characterized genes involved in its PWN-killing efficacy using transposon and PCR-targeted mutagenesis. First, by screening a miniTn10 transposon mutant library of strain AA4, we identified a mutant with greatly reduced nematocidal activity against *B. xylophilus*. We further analyzed the genomic site where the transposon was located and found a miniTn10 element that was inserted into an open reading frame (ORF) located between kilobase positions 3,956,912 and 3,955,545 (Supplementary Figure 4). This ORF was identified as the *sdaB* gene encoding the L-serine dehydratase beta subunit, indicating that the *sdaB* gene might be involved in the PWN-killing effect of AA4.

**FIGURE 5:**
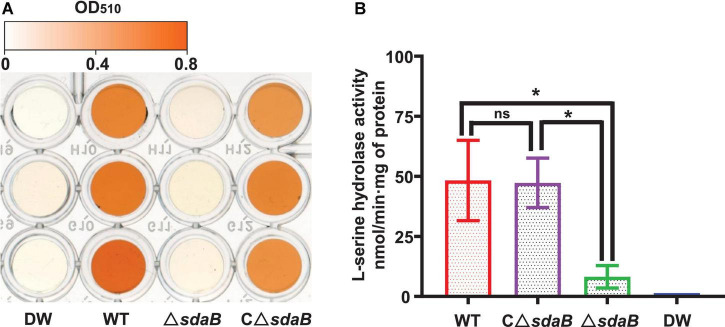
L-serine hydrolase activity of *E. ludwigii* AA4 and its *sdaB* gene knock-out mutant. **(A)** Colorimetrical quantification of pyruvate generated in a L-serine hydrolase activity assay. The color shown in each well of the microplate corresponds to the pyruvate concentration measured by a spectrophotometer at 520 nm. The values of optical density (OD) range from 0 (white) to 0.8 (orange). **(B)** Comparison of L-serine hydrolase activities of wild-type *E. ludwigii* AA4, its *sdaB* gene knock-out mutant, and AA4Δ*sdaB* complemented with a wild-type *sdaB* gene. DW, distilled water; WT, *E. ludwigii* AA4 wild-type strain; Δ*sdaB*, AA4 *sdaB* gene knock-out mutant; CΔ*sdaB*, AA4Δ*sdaB* complemented with a wild-type *sdaB* gene. The asterisks indicate that differences among the means represented by the columns are statistically significant (*P < 0.0001). Two-tailed *t*-test (GrapPad Prism 8) was used for the analysis.

We next created a knock-out mutant of the *sdaB* gene by employing the Red (λ, β, exo)-mediated DNA homologous recombination system. The L-serine hydrolase activity of the *sdaB* gene knock-out deletion mutant AA4Δ*sdaB* was significantly lower than that of the wild-type AA4 or the AA4Δ*sdaB* mutant strain complemented with the *sdaB* wild-type gene (Figure 5B), which indicated that the interruption of the *sdaB* gene affected the biosynthesis of L-serine dehydratase. Then, we compared the PWN-killing activities of the wild-type AA4 and AA4Δ*sdaB* by using the fluorescent staining-based nematocidal activity assay (Figure 2A). Nearly no fluorescence could be detected in the worms treated with AA4Δ*sdaB* cells or their extracts, which confirmed the loss of nematocidal activity of the miniTn10 insertional mutant. Complementation of the mutant with a recombinant plasmid harboring *sdaB* partially restored the PWN-killing activity (Figure 2A). These results were corroborated by the nematode mortality curves, which documented that the corrected death rate of the PWNs treated by AA4Δ*sdaB* was significantly lower than those incubated with wild-type AA4, while the mortality of nematodes exposed to the mutant strain complemented with the *sdaB* wild-type gene increased remarkably (Figure 2B). Therefore, *sdaB* gene product has a major impact on the nematocidal activity of *E. ludwigii* AA4.

**FIGURE 6:**
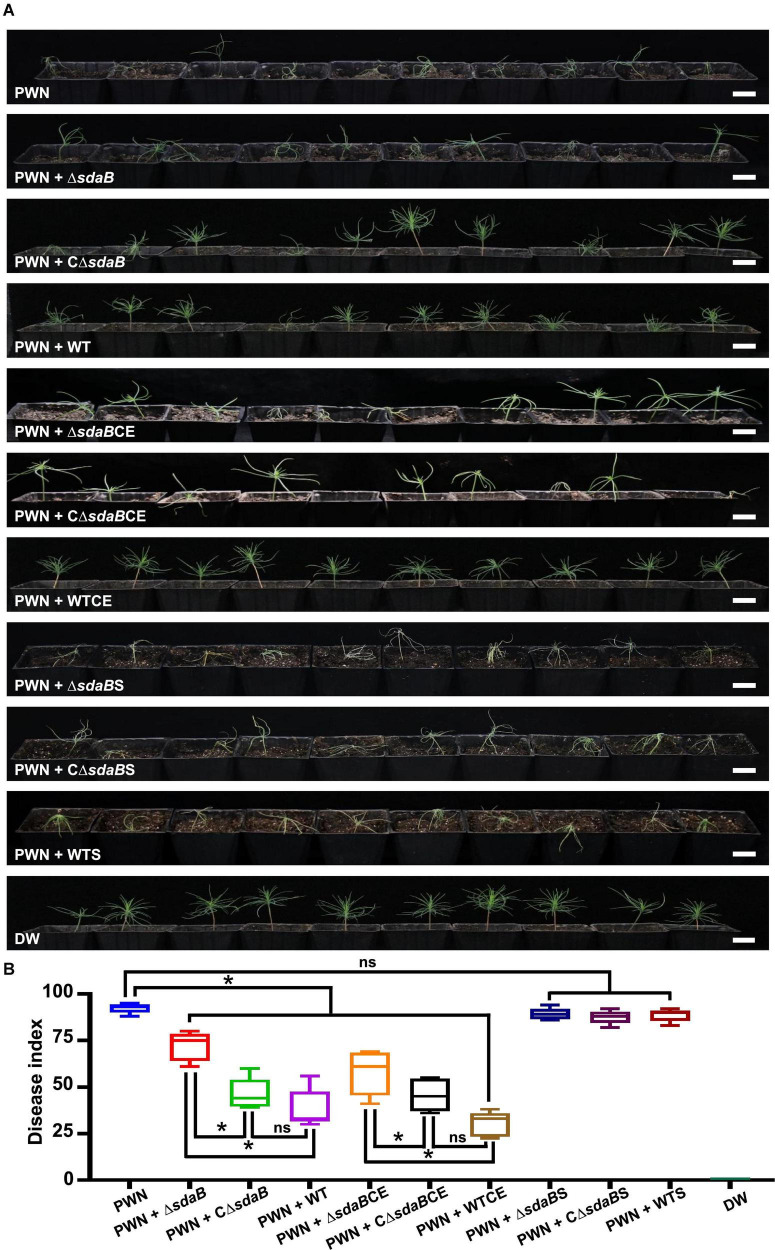
Disease-suppressing effect of *E. ludwigii* AA4 against PWD caused by *B. xylophilus*. **(A)** Biocontrol effect of *E. ludwigii* AA4 and its *sdaB* gene knock-out mutant against PWD on 1-month-old *P. sylvestris* seedlings. **(B)** Severity of PWD on 1-month-old *P. sylvestris* seedlings treated with PWN jointly with wild-type AA4 or AA4Δ*sdaB*. The horizontal bars within boxes represent the median. The tops and bottoms of boxes represent the 75th and 25th quartiles, respectively. The upper and lower whiskers extend from 75th quartiles to the maxima and from the 25th quartiles to the minima, respectively. The asterisks indicate that differences among the means represented by the boxes are statistically significant (*p ≤ 0.0345). Two-tailed *t*-test (GrapPad Prism 8) was used for the analysis. PWN, treatment with PWN alone; PWN + Δ*sdaB*, joint treatment with PWN and AA4Δ*sdaB*; PWN + CΔ*sdaB*, joint treatment with PWN and AA4CΔ*sdaB*; PWN + WT, joint treatment with PWN and wild-type AA4; PWN + Δ*sdaB*CE, joint treatment with PWN and AA4Δ*sdaB* cell extract; PWN + CΔ*sdaB*CE, joint treatment with PWN and AA4CΔ*sdaB* cell extract; PWN + WTCE, joint treatment with PWN and wild-type AA4 cell extract; PWN + Δ*sdaB*S, joint treatment with PWN and AA4Δ*sdaB* culture supernatant; PWN + CΔ*sdaB*S, joint treatment with PWN and AA4CΔ*sdaB* culture supernatant; PWN + WTS, joint treatment with PWN and wild-type AA4 culture supernatant; DW, treatment with distilled water (scale bars: 2 cm).

Then, we investigated the role of the *sdaB* gene product on the protective effect of *E. ludwigii* AA4 on *P. sylvestris* against PWD. Using a Pine-Nematode-Bacterium tripartite interaction system with 1-month-old *P. sylvestris* seedlings, a lower disease index was estimated when wild-type AA4 cells or their extracts were added to the system. Under these conditions, much less wilted and fewer chlorotic pine seedlings were observed compared to the control containing *B. xylophilus* alone (Figure 6B). We found more wilted and chlorotic seedlings when AA4Δ*sdaB* cells or their extracts were added instead of wild-type AA4 cells or their extracts. The number of diseased plants was reduced when the seedlings were treated with PWNs together with cells or cell extracts of the AA4Δ*sdaB* mutant strain complemented with the *sdaB* wild-type gene (Figure 6A). These findings were supported by disease severity analysis, where the disease index of plants treated with PWNs and AA4Δ*sdaB* mutant cells or cell extracts was significantly higher than that with PWNs treated with the cells or cell extracts of wild-type AA4 or the *sdaB* mutant complemented with the *sdaB* wild-type gene (Figure 6B). The indices of the PWN infected seedlings treated with cells or cell extracts of the AA4Δ*sdaB* mutant complemented with the wild-type *sdaB* gene and seedlings treated with PWNs plus the wild-type AA4 strain cells or cell extracts showed no significant differences. The culture supernatants of the wild-type AA4, the AA4Δ*sdaB* mutant strain, or AA4Δ*sdaB* mutant complemented with the *sdaB* wild-type gene not only exhibited nearly no nematocidal activity against *B. xylophilus in vitro*, but also failed to display biocontrol efficacy against PWD (Figure 6). The *sdaB* gene encoding the beta subunit of L-serine dehydratase, is highly related to the PWN-killing activity of *E. ludwigii* AA4 and may be a key molecular element controlling the interplay between strain AA4 and PWNs.

### Colonization of *E. ludwigii* AA4 on Needles of *P. sylvestris*

**FIGURE 7:**
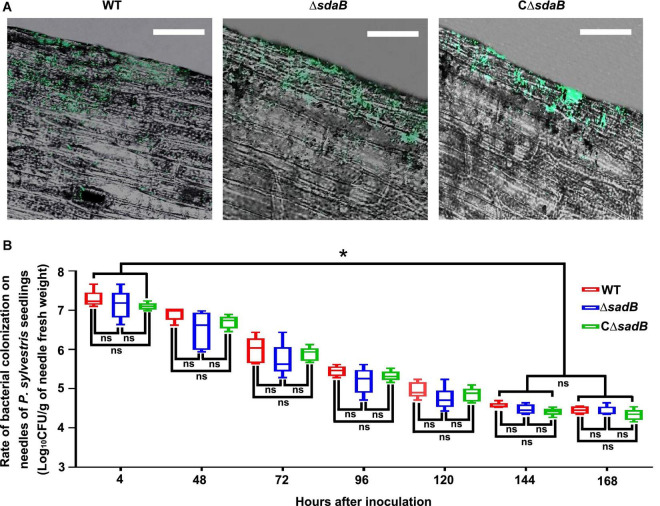
Colonization of *E. ludwigii* AA4 on needles of *P. sylvestris* seedlings. **(A)** Image analysis of needle colonization of green fluorescent protein (GFP)-labeled wild-type AA4, AA4 *sdaB* gene knock-out mutant, and AA4Δ*sdaB* complemented with a wild-type *sdaB* gene. These images were taken using a confocal laser scanning microscope at 48 h post inoculation (Scale bars: 20 μm). **(B)** The abundance of wild-type AA4, AA4 *sdaB* gene knock-out mutant, and AA4Δ*sdaB* complemented with a wild-type *sdaB* gene colonizing on the needles of *P. sylvestris* seedlings. WT, AA4 wild-type strain; Δ*sdaB*, AA4 *sdaB* gene knock-out mutant; CΔ*sdaB*, AA4Δ*sdaB* complemented with a wild-type *sdaB* gene. The asterisks indicate that differences among the means represented by the columns are statistically significant (*p ≤ 0.0041). Two-tailed *t*-test (GrapPad Prism 8) was used for the analysis.

Efficient colonization of plant surfaces and tissues is fundamental for successful inhibition of pathogens by biological control agents. To investigate the ability of *E. ludwigii* AA4 to colonize *P. sylvestris*, we sprayed the needles with a GFP-labeled transgenic strain of AA4. We observed by CLSM that strain AA4 was capable of adhering to the surface of *P. sylvestris* needles by forming robust biofilms (Figure 7A). Although there was a dramatical reduction of the abundance of AA4 cells sticking to the needles, from around 1.9 × 10^7^ CFU (colony-forming unit)/g (of pine needle fresh weight) 4 h after inoculation to 3.8 × 10^4^ CFU/g 144 h after inoculation, the colonization rates of AA4 at days 6 and 7 (2.8 × 10^4^ CFU/g) showed no significant difference (Figure 7B). This indicates that *E. ludwigii* AA4 can colonize the needles of *P. sylvestris*, presumably a precondition for its disease-controlling efficacy against PWD. AA4Δ*sdaB* cells were equally able to colonize pine needles compared to the wild-type AA4 (Figure 7A), demonstrating that the *sdaB* gene function is not needed for colonization. The biomass accumulation rate of both strains was at the same level along our 7-day-long experiment (Figure 7B). Therefore, *E. ludwigii* AA4 may be an efficient colonizer of pine needles, and that the eradication of *sdaB* gene did not affect its adherence to needles.

## Discussion

Compared to the control of phytopathogens in agroecosystems, forest disease suppression puts more emphases on the sustainable management of pests due to the vital functionality of forests in maintaining the balance and health of their ecosystems (Nascimento et al., 2015; Proenca et al., 2017; Khan et al., 2020; Ozair et al., 2020). To this end, biological control of forest pathogens, including the PWNs, by beneficial microbes have been receiving increasing attention (Nunes da Silva et al., 2015; Ponpandian et al., 2019; Proenca et al., 2019). A considerable number of nematocidal microbial strains suppressing *B. xylophilus* have been identified and characterized. These strains are, mainly, species of *Bacillus* (Crickmore, 2005; Niu et al., 2011; Park et al., 2020), *Streptomyces* (Kang et al., 2021), *Serratia* (Paiva et al., 2013; Nascimento et al., 2016; Abd El-Aal et al., 2021), and *Stenotrophomonas* (Huang et al., 2009; Ponpandian et al., 2019).

We selected a PWN-killing *Enterobacter ludwigii* strain, designated as AA4 from a large-scale screening of nematocidal activity and inhibition of PWD (Figures 1C, 3). Representatives of the genus *Enterobacter* are able to inhibit a wide range of phytopathogens (Demirci et al., 2000; Kageyama and Nelson, 2003; Windstam and Nelson, 2008; Del Barrio-Duque et al., 2019; Batyrova et al., 2020), including plant pathogenic nematodes, such as root-knot nematodes (Munif et al., 2000; El-Sayed et al., 2014; Oh et al., 2018). To our knowledge, *E. ludwigii* AA4 is the first *Enterobacter* strain described as a nematocidal agent suppressing *B. xylophilus*. Our findings expand the knowledge of the biocontrol potential of the genus *Enterobacter*, one of the most expanding bacterial taxa in recent decades (Taghavi et al., 2010; Ibort et al., 2018; François et al., 2021).

Accurate and rapid determination of the mortality of nematodes is the key to efficient screening and selection of microbial strains with nematocidal activity. In earlier studies on PPN-bacterium interactions, the killing rate of nematodes was estimated mainly by the morphology of the worms, where rigid nematodes were considered dead, while the curved ones were considered alive (Liu et al., 2016, 2019; Ponpandian et al., 2019; Qiu et al., 2019). Such criteria do not always work well. We found that a significant number of apparently rigid nematodes could still move after being touched with a needle, suggesting that they were still alive. In addition, counting dead nematodes under a microscope is time consuming and tedious.

We developed a visual PWN-killing activity DAPI dye exclusion which is faster and more reliable (Figure 2). DAPI passes slowly through intact cell membranes of living nematodes, and thus preferentially stains the dead nematodes. Once entering the cells and binding to the minor groove of A-T-rich regions of DNA, DAPI fluorescence is greatly enhanced (Atale et al., 2014). As a result, dead worms display intense fluorescent signals. A similar method was employed in a previous study, where the dead *Caenorhabditis elegans* nematodes were stained with the fluorophore Sytox Orange (Natalia et al., 2013; Rajamuthiah et al., 2015; Xie et al., 2020). Applying fluorescence staining in the identification of PWN-killing bacterial strains may be amenable to high throughput or automated screening for biocontrol agents against other plant pathogenic nematodes.

In present study, we observed that the PWNs treated with cell extracts of wild-type *E. ludwigii* AA4 displayed dramatic morphological defects associated with the accumulation of numerous vacuoles, recognized as a sign of reversible cell injury (RCI) and methuosis, resulting in the destruction of internal organs of *B. xylophilus* needed for absorbing nutrients, pathogenicity, and reproduction (Figure 4). Similar changes in morphology were also reported in *B. xylophilus* incubated with indoles and abamectin (Rajasekharan et al., 2017). Another plant pathogenic nematode, *Meloidogyne incognita*, the causal agent of root knot in several crops, showed bubbling in its body when meeting toxic compounds as well (Jang et al., 2016). A large number of vacuoles were present in the transgenic *C. elegans* with an ectopically expressed and activated *mek-1* gene (Koga et al., 2000). Intracytoplasmic vacuolation may commonly occur when nematodes live under stressful conditions, which can be triggered by fluid accumulation within the cytoplasm due to Ca^2+^ and water influx caused by RCI (Jurkowitz-Alexander et al., 1992; Basavappa et al., 1998; Rajasekharan et al., 2017). The death of *B. xylophilus* caused by AA4 might be attributed to methuosis induced by damage to the cell membrane caused by this nematocidal bacterial strain. The mechanisms underlying the PWN-killing function of *E. ludwigii* AA4 remains to be determined.

Some mechanisms of nematocidal activity against *B. xylophilus* have been recently documented, such as parasitization (Proenca et al., 2017; Ponpandian et al., 2019), production of toxins (Abd El-Aal et al., 2021; Kahn et al., 2021), antibiotics and destructive enzymes (Yang et al., 2007; Huang et al., 2009) or competing for nutrients (Proenca et al., 2019), and inducing systemic resistance of plants (Liang et al., 2019; Han et al., 2021). The molecular mechanisms underlying these PWN-killing effects are still largely unknown. In this work, we demonstrate that the *sdaB* gene encoding the beta subunit of L-serine dehydratase is involved in the nematocidal activity of *E. ludwigii* AA4 against PWNs. The deletion of this gene caused significant impairment in both L-serine hydrolase activity (Figure 5) and ability to inhibit PWN (Figures 2, 6), but not the growth of *E. ludwigii* AA4 *in vitro* or *in planta* (Supplementary Figure 2). L-serine dehydratase activity is widely found in diverse organisms. It specifically deaminates L-serine to produce pyruvate and ammonia (Xu et al., 2011; Grant, 2012; Thoden et al., 2014), and plays a critical role in maintaining amino acid homeostasis during shifts in nutrient availability (Velayudhan et al., 2004; Zhang et al., 2010; Chen et al., 2012). Until now, little or no data about the contribution of L-serine dehydratase to microbial nematocidal activities has been reported. L-serine is an important biomolecule, and excessive amounts of intracellular serine inhibit the biosynthesis of other amino acids (Zhang et al., 2018, 2019; Révora et al., 2020; Wang et al., 2020) essential to the production of certain bacterial toxins *via* the non-ribosomal peptide synthetase (NRPS) pathway (Cao et al., 2008; Soeriyadi et al., 2021).

Here, we assume that the *sdaB* gene product might control *B. xylophilus* by regulation of potential anti-nematode compounds in *E. ludwigii* AA4. Considering the tight relationship between L-serine and methylation reactions (Kalhan and Hanson, 2012; Datta et al., 2016; Bignell et al., 2018; Kriner and Subramaniam, 2020), such modulation may be linked to bacterial epigenetics. We noticed that the supernatant of strain AA4 cell culture has almost no effect (Figures 2, 6), which may be due to the low concentration of nematocidal compounds in the broth or suggests that the PWN-killing efficacy of AA4 might be contact-dependent. In future work, we will more deeply analyze the function of the *sdaB* gene in the nematocidal activity of *E. ludwigii* AA4.

Colonization of plant hosts is a necessary precondition for efficient pathogen inhibition by microbial biocontrol agents (Vurukonda et al., 2018; Niu et al., 2020; Zhang J. et al., 2020; Vandana et al., 2021). *E. ludwigii* AA4 was capable of colonizing the needles of *P. sylvestris* by forming sturdy biofilms (Figure 7), indicating its high affinity to pine needles, despite its presumed origin from herbaceous host plants. This finding underlines the great adaptability of the genus *Enterobacter*, which appears to be highly adaptable to diverse environments (Shoebitz et al., 2009; Singh et al., 2017; Shastry et al., 2020; Ranawat et al., 2021). These features may facilitate the development of an AA4-based biopesticide against *B. xylophilus*. Identification of genes and proteins involved in resistance to pests and pathogens is of long-term interest because such molecules represent the next generation of targets for creation of nematode resistant plants through genetic engineering or targeted mutagenesis. Our findings also pave the way for introducing agriculture biocontrol agents in forest disease management.

## Data Availability Statement

The data presented in the study are deposited in the Genbank repository, accession numbers CP018785, OM883853, OM899759-OM899796, OM899810, OM900025, and OM900026.

## Author Contributions

BN and RB designed the project. YZ and BN wrote the manuscript. BN, RB, ZY, RS, and HS revised the manuscript. YZ and ZY performed the experiments. YZ, SW, DW, and BN analyzed the data. YC created the random transposon library. HW, HY, JP, and MP performed the *in planta* biocontrol assays. All authors listed have made a substantial, direct, and intellectual contribution to the work, and approved it for publication.

## Conflict of Interest

The authors declare that the research was conducted in the absence of any commercial or financial relationships that could be construed as a potential conflict of interest.

## Publisher’s Note

All claims expressed in this article are solely those of the authors and do not necessarily represent those of their affiliated organizations, or those of the publisher, the editors and the reviewers. Any product that may be evaluated in this article, or claim that may be made by its manufacturer, is not guaranteed or endorsed by the publisher.

## Footnotes

1
http://staden.sourceforge.net/

2
https://blast.ncbi.nlm.nih.gov/

3
http://rdp.cme.msu.edu

## Supplementary Material

Supplementary Figure 1 The corrected mortality of 43 efficient pine wood nematode (PWN)-killing bacterial strains. *Bacillus pumilus* YLT40 and *Paenibacillus polymyxa* M-1 were used as positive and negative controls, respectively. Asterisk indicates that differences among the means represented by the columns are statistically significant (**p* < 0.0001). Two-tailed *t*-test (GrapPad Prism 8) was used for the analysis.

Supplementary Figure 2 Growth curves of wild-type *E. ludwigii* AA4, its *sdaB* gene knock-out mutant, and AA4Δ*sdaB* complemented with a wild-type *sdaB* gene. WT, *E. ludwigii* AA4 wild-type strain; Δ*sdaB*, AA4 *sdaB* gene knock-out mutant; CΔ*sdaB*, AA4Δ*sdaB* complemented with a wild-type *sdaB* gene; LB, sterile Luria-Bertani liquid medium.

Supplementary Figure 3 Disease ranks of the pine wilt disease (PWD) caused by *B. xylophilus* under greenhouse conditions on day 9 after inoculation (scale bars: 20 μm). In rank 0, stems and needles are green and plump. In rank 1, yellowish speckles are presented on stems, and stems and needles appear slightly shriveled. In rank 2, stems turn yellow and shriveled, and less than 25% needles are partially wilted. In rank 3, stems turn yellow and shriveled, among which 25–50% of needles are partially wilted. In rank 4, stems turn brownish and completely shriveled, and more than 50% of needles are completely wilted and turn yellowish. W, the whole views of *P. sylvestris* seedlings; T, the top views of *P. sylvestris* seedlings; S, the stems of *P. sylvestris* seedlings; N, the needles of *P. sylvestris* seedlings.

Supplementary Figure 4 The location of miniTn10 transposon on the genome sequence of *E. ludwigii* AA4. The site of miniTn10 transposon insertion is indicated by a black triangle.

Supplementary Table 1 Corrected mortality rates for the 43 efficient pine wood nematode (PWN)-killing strains.
