# Supplementary material for: Gene sdaB Is Involved in the Nematocidal Activity of Enterobacter ludwigii AA4 Against the Pine Wood Nematode Bursaphelenchus xylophilus
Source: Front Microbiol. 2022 May 6;13:870519. doi: 10.3389/fmicb.2022.870519 (PMC9121001; doi:10.3389/fmicb.2022.870519)
Supplement: Supplementary Figure 1 — The corrected mortality of 43 efficient pine wood nematode (PWN)-killing bacterial strains. Bacillus pumilus YLT40 and Paenibacillus polymyxa M-1 were used as positive and negative controls, respectively. Asterisk indicates that differences among the means represented by the columns are statistically significant (*p < 0.0001). Two-tailed t-test (GrapPad Prism 8) was used for the analysis. [file Table_1.docx]

**Table. S1 Corrected mortality rates for the 43 efficient PWN-killing strains**

| **Number** | **Code** | **Taxonomy** | **Corrected mortality rate (%)** | **GenBank**  **Accession number** |
| --- | --- | --- | --- | --- |
| 1 | AA4 | *Enterobacter ludwigii* | 98.3 | CP018785 |
| 2 | LC257 | *Enterobacter* sp. | 94.1 | OM899785 |
| 3 | LC246 | *Enterobacter* sp. | 92.2 | OM899784 |
| 4 | LC243 | *Enterobacter* sp. | 90.1 | OM899781 |
| 5 | LC228 | *Enterobacter* sp. | 89.5 | OM899780 |
| 6 | LC244 | *Enterobacter cloacae* | 90.9 | OM899782 |
| 7 | LC245 | *Enterobacter cloacae* | 90.0 | OM899783 |
| 8 | LC021 | *Bacillus* sp. | 94.1 | OM899759 |
| 9 | LC075 | *Bacillus* sp. | 93.9 | OM899761 |
| 10 | LC192 | *Bacillus* sp. | 92.9 | OM899776 |
| 11 | LC080 | *Bacillus* sp. | 92.8 | OM899762 |
| 12 | LC165 | *Bacillus* sp. | 92.6 | OM899770 |
| 13 | LC023 | *Bacillus* sp. | 90.8 | OM899760 |
| 14 | LC168 | *Bacillus subtilis* | 94.4 | OM899771 |
| 15 | LC170 | *Bacillus subtilis* | 94.2 | OM899772 |
| 16 | LC173 | *Bacillus subtilis* | 91.5 | OM899773 |
| 17 | LC154 | *Bacillus pumilus* | 91.6 | OM899768 |
| 18 | LC152 | *Bacillus pumilus* | 91.4 | OM899767 |
| 19 | LC155 | *Bacillus pumilus* | 91.1 | OM899769 |
| 20 | LC090 | *Bacillus cereus* | 92.4 | OM899763 |
| 21 | LC109 | *Bacillus cereus* | 91.6 | OM899764 |
| 22 | LC139 | *Bacillus firmus* | 92.3 | OM899766 |
| 23 | LC138 | *Bacillus firmus* | 91.9 | OM899765 |
| 24 | LC185 | *Bacillus thuringiensis* | 91.3 | OM899775 |
| 25 | LC184 | *Bacillus thuringiensis* | 91.0 | OM899774 |
| 26 | LC015 | *Bacillus anthracis* | 96.9 | OM883853 |
| 27 | LC201 | *Chryseobacterium* sp. | 93.8 | OM899777 |
| 28 | LC211 | *Chryseobacterium* sp. | 93.6 | OM899778 |
| 29 | LC213 | *Chryseobacterium* sp. | 92.5 | OM899779 |
| 30 | LC300 | *Pseudomonas* sp. | 90.8 | OM900025 |
| 31 | LC303 | *Pseudomonas* sp. | 90.3 | OM900026 |
| 32 | LC305 | *Pseudomonas* sp. | 89.4 | OM899790 |
| 33 | LC306 | *Pseudomonas* sp. | 89.1 | OM899791 |
| 34 | LC310 | *Pseudomonas* sp. | 87.0 | OM899792 |
| 35 | LC291 | *Pseudomonas* sp. | 86.9 | OM899810 |
| 36 | LC294 | *Pseudomonas geniculata* | 92.0 | OM899788 |
| 37 | LC297 | *Pseudomonas geniculata* | 91.9 | OM899789 |
| 38 | LC408 | *Stenotrophomonas maltophilia* | 92.2 | OM899796 |
| 39 | LC404 | *Stenotrophomonas maltophilia* | 90.5 | OM899794 |
| 40 | LC407 | *Stenotrophomonas maltophilia* | 90.1 | OM899795 |
| 41 | LC347 | *Stenotrophomonas* sp. | 88.9 | OM899793 |
| 42 | LC286 | *Paenibacillus* sp. | 87.9 | OM899786 |
| 43 | LC288 | *Pantoea agglomerans* | 90.0 | OM899787 |
